# When Pictures Waste a Thousand Words: Analysis of the 2009 H1N1 Pandemic on Television News

**DOI:** 10.1371/journal.pone.0064070

**Published:** 2013-05-17

**Authors:** Westerly Luth, Cindy Jardine, Tania Bubela

**Affiliations:** 1 Department of Public Health Sciences, School of Public Health, University of Alberta, Edmonton, Canada; 2 Center for Health Promotion Studies, School of Public Health, University of Alberta, Edmonton, Canada; Dana-Farber Cancer Institute, United States of America

## Abstract

**Objectives:**

Effective communication by public health agencies during a pandemic promotes the adoption of recommended health behaviours. However, *more* information is not always the solution. Rather, attention must be paid to *how* information is communicated. Our study examines the television news, which combines video and audio content. We analyse (1) the content of television news about the H1N1 pandemic and vaccination campaign in Alberta, Canada; (2) the extent to which television news content conveyed key public health agency messages; (3) the extent of discrepancies in audio versus visual content.

**Methods:**

We searched for “swine flu” and “H1N1” in local English news broadcasts from the CTV online video archive. We coded the audio and visual content of 47 news clips during the peak period of coverage from April to November 2009 and identified discrepancies between audio and visual content.

**Results:**

The dominant themes on CTV news were the vaccination rollout, vaccine shortages, long line-ups (queues) at vaccination clinics and defensive responses by public health officials. There were discrepancies in the priority groups identified by the provincial health agency (Alberta Health and Wellness) and television news coverage as well as discrepancies between audio and visual content of news clips. Public health officials were presented in official settings rather than as public health practitioners.

**Conclusion:**

The news footage did not match the main public health messages about risk levels and priority groups. Public health agencies lost control of their message as the media focused on failures in the rollout of the vaccination campaign. Spokespeople can enhance their local credibility by emphasizing their role as public health practitioners. Public health agencies need to learn from the H1N1 pandemic so that future television communications do not add to public confusion, demonstrate bureaucratic ineffectiveness and contribute to low vaccination rates.

## Introduction

The H1N1 pandemic of 2009 challenged the capacity of public health agencies worldwide to respond to rapidly evolving information about the nature, seriousness and extent of the threat. On June 11, 2009, the World Health Organization (WHO) raised the influenza pandemic alert to Phase 6, the highest possible level [1], because of the potential for geographic spread, but maintained that the severity of the pandemic would be “moderate” [2]. The discordance between a highest level alert and a “moderately” severe pandemic captured the confusion generated by public health agency communications.

Effective communication by public health agencies during a pandemic is critical because many sources bombard the public with contradictory information, some reputable and some not [3]. To support informed health decisions, communications need to be accessible and meaningful, recognizing that during pandemics members of the public may make decisions largely based on emotion [4]. If the intended result is to enhance the adoption of recommended health behaviours, providing *more* information is not always the solution. Rather, public health agencies need to pay attention to *how* the information is communicated. Effective communication requires a sophisticated understanding of both the selection of media used by the public to inform health decisions and the inherent advantages and limitations of the chosen medium.

In this context, we analysed official communications in a dominant medium – television – in response to the 2009 H1N1 pandemic and vaccination program in Alberta, Canada. We examined television news coverage because it remains a significant source of health information for the public internationally, despite the rise of the Internet­ [3], [5]. Television is under-studied compared to print sources of health information [6], [7]. Unlike print, television combines images, graphics and video footage with audio from news anchors, reporters and interviewees. Our analysis, therefore, has three aims: (1) to describe the content of television news about the H1N1 pandemic and vaccination campaign in Alberta, Canada; (2) to analyse the extent to which television news content conveyed key public health agency messages; and (3) to analyse the extent of discrepancies, and even direct contradictions, between audio and visual content.

### Alberta Context for the H1N1 Pandemic and Media Communications

In Canada, provinces are responsible for the provision of health services. At the provincial level in Alberta, there was overlapping jurisdiction in the organization and implementation of the provincial pandemic response strategy amongst Alberta Health and Wellness (AHW), which provided policy direction; Alberta Health Services (AHS) responsible for delivery of health services; and the Alberta Emergency Management Agency (AEMA), which coordinated the response to the pandemic [8]. The key actors during the time were Mr. Ron Liepert, Alberta’s Minister of Health and therefore head of AHW; Dr. Andre Corriveau, Alberta’s Chief Medical Officer of Health, who provided public health expertise to AHW; and the Medical Officers of Health, Dr. Gerry Predy and Dr. Judy Macdonald, who provided public health expertise to AHS within provincial health zones. AEMA did not feature in television coverage.

Setting policy at the federal level and appearing in media coverage of the H1N1 pandemic were Dr. David Butler-Jones, Canada’s Chief Public Health Officer of Canada and head of the Public Health Agency of Canada (PHAC) and The Honourable Leona Aglukkaq, Minister of Health and head of Health Canada. Federal and provincial governments were all involved in the organization and implementation of the H1N1 pandemic response in Alberta.

The timeline of events and responses to the pandemic are outlined in Table 1. The first H1N1 fatality in Alberta was reported on May 8, 2009 by which time 42 Albertan cases had been confirmed. In August, 2009 the Government of Canada announced its intention to order 50.4 million H1N1 vaccine doses, and in October, Health Canada approved the H1N1 vaccine. As the primary health mitigation strategy, Alberta opened vaccination clinics to any Albertan who wanted to get vaccinated on October 26, 2009 [9]. There was an immediate rush to the vaccination clinics, which were overwhelmed by demand. The clinics were closed five days later because of a projected vaccine shortage caused by an unanticipated shortfall in the promised supply [9]. They reopened on November 5, 2009, but Alberta’s public health agency communications shifted to identifying priority groups and the sequence in which they were to be vaccinated [9]. The first priority group was children 6 months to 5 years old [9]. Over time the clinics opened to other members of priority groups (Table 1). By November 23 the vaccine shortage was resolved and the vaccination clinics were opened to all Albertans over 6 months old [9]. By the end of the pandemic and vaccination campaign, 71 deaths in Alberta were attributed to H1N1 complications [9], and only 36.6% of Albertans had been vaccinated - one of the lowest provincial rates in Canada [10].

**Table 1:** Timetable of key events in Alberta of the H1N1 pandemic between March 29, 2009 and May 31, 2010*.

Date	Event	
March 29, 2009	WHO identifies first case of H1N1 in Mexico.	
April 13	WHO identifies first death attributed to H1N in Mexico.	
May 8	Alberta Public Health agencies report first H1N1 fatality in Alberta. 42 cases are confirmed in Alberta.	
August 6	Canadian government announces intention to order 50.4 million doses of H1N1 vaccine.	
October 21	Health Canada approves H1N1 vaccine.	
October 26	Alberta vaccination clinics open.	
October 31	Alberta vaccination clinics temporarily close due to a vaccine shortage.	
November 5	Alberta vaccination clinics reopen to: children 6 months to 5 years old;	
November 6	pregnant women;	
November 10	parents/caregiver of infants under 6 months; children under 10 with chronic conditions;	
November 12	people with chronic conditions aged 10–17 and 55 to 64;	
November 13	people with chronic conditions aged 45 to 55;	
November 14	people between 18 and 44 with chronic conditions; household contacts of those who cannot be immunized; frontline health care workers;	
November 16	all health care workers, first responders, provincial corrections inmates, provincial peace officers and essential service workers;	
November 17	seniors over 75 and spouses or partners of any age;	
November 19	seniors over 65 and spouses or partners of any age;	
November 20	children under 18 and immediate family members and cohabitating caregivers;	
November 23	all members of the public.	
May 31, 2010	71 H1N1 fatalities confirmed in Alberta.	

* Adapted from Alberta Health and Wellness and Alberta Health Services [9].

In addition to vaccination, other key health mitigation strategies in Alberta’s plans for the H1N1 pandemic were public education and anti-viral medication [11]. Anti-virals were not considered preventative. However, the public education campaign, modified to reflect the epidemiology of H1N1, was supposed to educate Albertans about “how to protect themselves and others, how to avoid spreading the disease when they are sick and basic emergency preparedness” [11]. However, immunization was expected to be the “most effective strategy to prevent pandemic influenza” [11]. While hand hygiene and other behavioural measures appeared in Alberta’s pandemic plan, none were privileged to the same extent as vaccination. For example, public education material stated: “*Next to immunization*, the single most important way to prevent influenza is frequent and thorough hand cleaning” [12] (emphasis added).

Alberta’s pandemic plan identified the media as central to the public education campaign [11]. Officials were to use “existing tools, techniques and processes to communicate to the public through the media” [11] with the implicit assumption that public health agencies could control media content. However, while media outlets can be effective partners for public health agencies during pandemics, the plan failed to recognize that the news media have goals, needs and challenges that will not always coincide with those of public health agencies. The news media operate in a highly competitive, fast-paced environment, which drives journalists to privilege some news stories over others [13]. To attract an audience, journalists preferentially cover dramatic, alarming events over on-going health concerns [3], [6]. Thus conflicts and aberrations are dominant news frames [6]. Frames are “interpretative packages and storylines that help communicate why an issue might be a problem, who or what might be responsible and what should be done” [14], [15]. They are used to simplify complex issues by lending greater weight to certain considerations and arguments over others; frames are an unavoidable reality of the communication process surrounding health issues and capture the attention of the public.

It is evident from reviews of Alberta’s response to the pandemic that media communications became a significant challenge throughout the pandemic, especially the negative response generated by the confused vaccination rollout [8]. One post-pandemic recommendation that emerged was to “educate” key media workers about the need for “informed and responsible reporting during a potentially serious pandemic” [15]. However, recommendations in this vein displace the responsibility of public health officials to understand the media environment, an issue we address here with respect to a prominent media format, television news.

## Methods

We obtained news clips from the CTV online video archive because it represented a readily accessible and comprehensive coverage set from the pre-eminent television news media provider in Alberta during the pandemic. CTV was the most watched local evening news program in Northern Alberta with 145,000 viewers [16], representing approximately 10% of the total population [17]. Competitor evening news programs on other Canadian networks, Global TV and CBC, had 107,000 and 11,000 viewers [16], respectively. CTV’s local evening news in Calgary had an average of 86,000 viewers [18], making it a top local news source in Southern Alberta.

### Coding Strategy and Inter-Coder Reliability

We searched for “swine flu” and “H1N1” in local English news broadcasts and retrieved 24 clips from Edmonton and 23 clips from Calgary (Alberta’s two largest cities, located in northern and southern Alberta, respectively) for a total of 2.14 hours of footage during the peak period of coverage from March to November 2009 (Table S1). Author, WL, coded the audio and visual content from each clip in NVivo™ 9 [19] using the codebook developed in consultation with TB (Tables S2, S3). The development of the codebook was an iterative process [20], involving the development of separate codebooks for the audio and visual content as we viewed and analysed the clips. We did not use an *a priori* coding frame, rather, the codes emerged from the data [20]. We added new codes to the codebook as these appeared in the clips until no new codes emerged. In the second iteration, WL re-examined previously analysed audio and visual content to ensure all clips were analysed for all codes in the codebook.

A second, experienced media coder was trained in the use of codebook for both audio and visual content to ensure validity of the developed codes and the reliability of their application to the clips. Following discussion and clarification of the codebook, the second coder independently coded 10% of the clips; the clips were selected using a random number generator. We calculated percent agreement and Kappa scores using the coding comparison query in NVivo™ 9. The percent agreement for audio content codes was between 88% and 100%; Kappa scores were between 0.76 and 1.00, with only 3 scores below 0.81. The percent agreement for video content codes was between 94% and 100% and Kappa scores were between 0.76 and 1.00, with only 2 scores below 0.81. All Kappa scores were therefore above 0.60, indicating “substantial” agreement [21]. McHugh [22] also suggests that percent agreement higher than 80% and Kappa scores higher than 0.60 indicate adequate agreement among the coders.

### Coding of Audio and Visual Content

First, we transcribed the audio from news anchors, reporters and interviewees. Where available, we recorded the names and positions of the individuals speaking. Coding of the audio transcripts captured both the subject-matter (e.g., long queues at vaccination clinics, referred to here and in media reports as line-ups) and the sentiment conveyed (the most dominant of which was frustration) (Table S2). Coding of audio content then identified specific groups (e.g., pregnant women); important events (e.g., temporary clinic closures, vaccine shortages, new cases of H1N1); and general themes (e.g., criticism of the government, individual vaccination decision-making).

**Figure 1:**
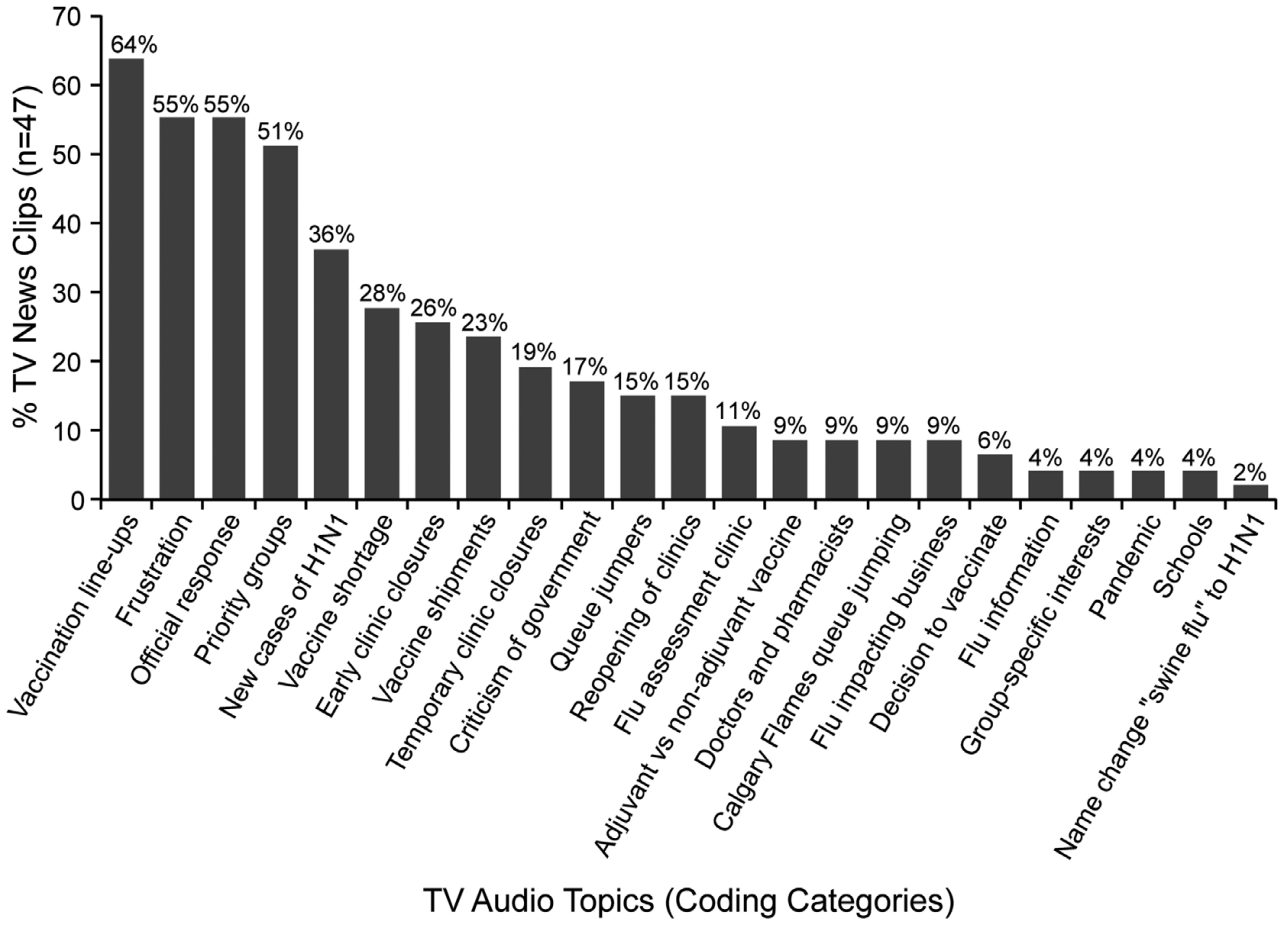
Main topics in news coverage. Percentage coverage of main topics mentioned in 47 CTV news clips of the 2009 H1N1 pandemic in Alberta. The most common topics were: the line-ups at vaccination clinics, the public’s frustration with the vaccination campaign, the official response to criticism of the vaccination campaign and the identification of priority groups. Key public health messages like H1N1 symptoms; information on H1N1 and its transmission; and vaccine safety received less attention in the news coverage.

WL then constructed a detailed written description of the video content. This description was verified through discussion with the second coder, who viewed the video clips concurrently with the written description in NVivo™ 9. The second coder, while given the opportunity to do so, made no alterations to the description. Coding of visual content identified the subject-matter of images (e.g., individuals being vaccinated and clinic line-ups); described the individuals portrayed (e.g., gender, approximate age, ethnicity, specific features such as pregnancy and apparel); and described the location (e.g., official press conferences, hospital or clinic setting, government buildings). After the coding of the audio and visual content of the television news clips was completed, we then clustered the codes into broader themes (Figure 1, Tables S2, S3). Note that segments of audio or visual content could be coded more than once. We also described the content of static information screens and graphic imagery/logos used in the news footage (Table S4).

### Analysis of Discrepancies in Audio and Visual Content

We re-examined the original footage using NVivo™ 9 [19] alongside the transcript of audio content and the description of the video content with the goal of noting discrepancies between audio and visual content and if such discrepancies were minor or major. Minor discrepancies occurred when there was concordance between visual and audio content in the topic but factual inaccuracies in specific details (e.g., footage of a clinic when audio content referred to a different clinic). Major discrepancies involved misrepresentations of important public health messages (e.g., visual footage of seemingly healthy, non-priority individuals in line-ups or being vaccinated when audio targeted vulnerable groups for vaccination).

### Semi-Structured Interviews

Finally our analyses were augmented by semi-structured interviews with five Canadian science and health journalists about their experience in covering the H1N1 pandemic, generally, and their impressions of the television news coverage, specifically. TB conducted the interviews (four by phone and one in-person) with three print media journalists (two national and one Alberta-based), one national radio broadcaster and one health journalist for Alberta television news, who was temporarily employed as a media public relations specialist at a research university. Interviewees were selected based on their extensive involvement with the Canadian Science Writers Association and history of coverage of health and science stories. Interviews addressed media coverage of the pandemic from first reports of the outbreak through to a post-pandemic assessment. Interviews explored, *inter alia*, impressions of public health agency communications throughout the pandemic; feedback on media coverage from the public; clarity and accuracy of media coverage; specific issues relevant to television coverage; and the potential for confusion caused by conflicting messages in media coverage. We used the transcribed interview transcripts to validate our findings about the nature of health journalism during a pandemic and the interactions between journalists and public health officials. Statements made by the journalists supported our conclusions; the interviews added to our understanding of the constraints under which journalists operate during a rapidly evolving public health news story.

### Ethics Statement

Ethics approval for the interviews was received from the Education, Extension, Augustana and Campus Saint-Jean Research Ethics Board (EEASJ REB) at the University of Alberta. Participant media experts provided their written informed consent to participate in this study through a semi-structured interview.

## Results

### News Clip Coding: Events and Themes

The main events covered by the CTV news coverage are summarized in Table 1. The events that dominated the news were the opening and closing of vaccination clinics. The dominant theme on CTV news was the ‘back and forth’ between the public, frustrated with long line-ups at vaccination clinics in Calgary, Edmonton, Red Deer and nearby rural communities and the government officials who responded to public outrage (Figure 1).

Despite the global scale of H1N1, the ‘pandemic’ nature of the H1N1 outbreak (severity and geographic scope) was highlighted in only 8% of clips. Local news focused on local events and concerns, including short bursts of coverage on controversial events. For example, television news highlighted a story about the Calgary Flames hockey team and family members being allegedly given the vaccine at a private clinic organized by Alberta Health Services while priority group members waited in line, thereby jumping the queue.

### Public Health Agency Communications versus Television Coverage

While Public Health Agencies emphasized the importance of vaccination as the primary prevention strategy, they also communicated information about other preventive measures as well as factual information about the disease [11]. However, important information regarding the disease (e.g., symptoms and transmission) and the vaccine (e.g., safety concerns and vaccine type) were covered in only 9% and 15% of clips, respectively.

### Minor Discrepancies between Audio and Visual Content

Minor discrepancies occurred in 66% of news clips. Episodes of minor discrepancies included the re-use of line-up footage from day to day, showing clinics not discussed in concurrent audio (45% of minor discrepancies); using interviews when discussing events elsewhere or on different days (19% of minor discrepancies); the misidentification of locations (10% of minor discrepancies); and showing the wrong name for a speaker, such as a public health official (6% of minor discrepancies).

### Public Health Agency Priority Groups: A Major Source of Discrepancies

Once it became evident that there was a vaccine shortage and that steps would have to be taken to prioritise groups to receive the vaccine, the Public Health Agencies outlined priority groups. Table 1 outlines priority groups targeted November 5–20, 2012, and Table 2 outlines the relative priority for different classes of Albertans to receive vaccination. Table 2 details the discrepancies between the priority groups identified by Alberta Health and Wellness (AHW) [11] and both audio and visual content of news clips. The reporting of priority groups comprised the largest class of major discrepancies between public health messages and television news content as well as between audio and visual content (47% of clips).

**Table 2:** Comparison of Alberta Health and Wellness priority groups and groups represented in television audio and visual content.

Priority	Alberta Health and Wellness*	Audio Content**	Visual Content**
1 - High	Adults and children with: cardiac or pulmonary disorders;diabetes mellitus and other metabolic disorders; cancer;immunodeficiency or immunosuppression; renal disease; anemiaor hemoglobinopathy; conditions compromising the managementof respiratory secretions; conditions treated with acetylsalicylicacid for a long time; residence in nursing homes, lodgesand other chronic care facilities; chronicallydisadvantaged living situations	Chronically ill children under 10 yearsold (4); Chronically ill seniors under 65 (4)	
	Pregnant women	Pregnant women (2)	Pregnant women (5)
2	Children six months to less than five years old	Young children (1); infants (6)	Young children and infants (4)
	People residing in remote and isolated settings or communities	People residing in remote communities (5)	
	Health care workers	Health care workers (5)	Health care workers (5)
3	Household contacts and care providers of: infants less thansix months old; persons who are immunocompromised	Parents of babies under six months (5);caregivers for high-risk individuals (7)	
4	Children five to 18 years old		Children 7–14 (3)
	First responders		
	Poultry and Swine workers		
	Adults 19 to 64 years old		Adults 19–64 years old (1)
5 - Low	Adults 65 of age and over	Seniors (3)	Seniors (2)

* Adapted from Alberta Health and Wellness [11].

** Bracketed numbers indicate the rank order of the appearance of priority groups in audio and video content, with 1 being most frequent. Only children six months to less than five years old, seniors, health care workers and pregnant women were identified in both modalities.

**Figure 2:**
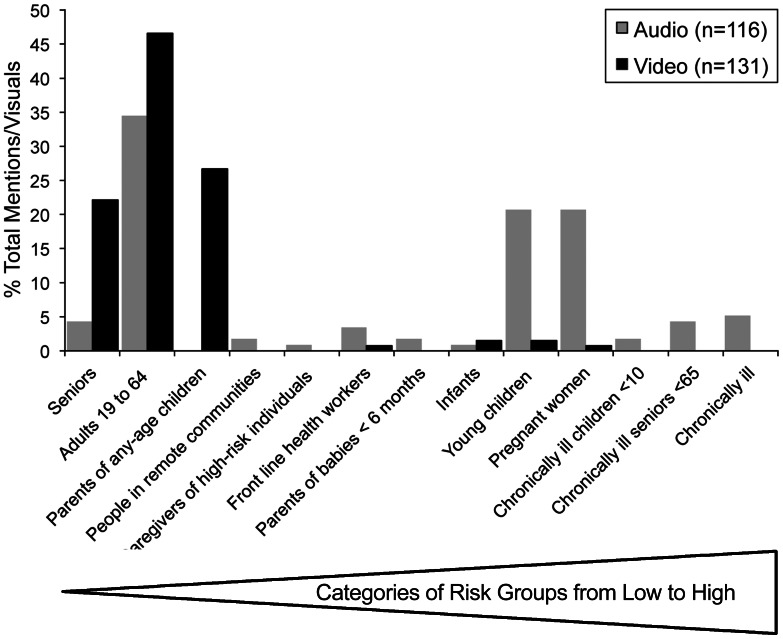
Comparison of vaccination priority groups in audio and visual content. Percentage of total mentions in audio content (n = 116) and visuals in video content (n = 131) of priority groups identified by Alberta Health and Wellness. The audio and visual content did not completely match the official priority groups. More groups and more high-priority groups were identified in the audio content. Low priority adults dominated the visual content. The highest priority groups were virtually absent from the visual content. Mid-level priority groups (e.g. people living in remote communities, infants, caregivers of high-risk individuals and front-line health care workers) were poorly represented in both modalities.

Of the priority groups, young children (34% of clips), pregnant women (32% of clips) and the chronically ill (21% of clips) dominated audio content. Seniors not resident in chronic care facilities were considered by AHW to be a low priority group but were shown in 30% of video clips (Figure 2).

Seniors were the third most commonly interviewed age category (30% of interviewees) after healthy-appearing adults (42%) and parents who appeared with children (35%). Despite mention as a priority group in 31% of clips, pregnant women were shown only once. Young children were defined by AHW as being from six months to 5 years old, but in news clips, age groups were rarely mentioned. No individuals were identified as chronically ill. In contrast, healthy-appearing adults, for example businessmen in shirt sleeves and seniors dominated visual content; 99% of individuals interviewed and 94% of those shown being vaccinated were not obvious members of a priority group.

In addition, the Public Health Agency of Canada (PHAC) prioritized vaccination for Aboriginal peoples because many already fit within other priority group criteria, especially those living in remote regions of Canada [11]. PHAC’s lead in prioritizing Aboriginal Peoples was followed by AHW [11]. Despite this focus, Aboriginal individuals only appeared three times in the visual content of CTV news, and reporters interviewed only one Aboriginal person. Aboriginal people were never identified as a priority group in the audio content.

### Spokespeople

Public health officials received 7.6% of total audio airtime and 8.6% of total video airtime. Provincial officials included Alberta Health and Wellness Minister Ron Liepert (34% of clips), Senior Medical Officer of Health, Dr. Gerry Predy (23% of clips), Alberta’s then Chief Medical Officer of Health, Dr. Andre Corriveau (15% of clips) and Calgary Zone Medical Officer of Health, Dr. Judy MacDonald (13% of clips). Federal officials, such as Canada’s Chief Medical Officer of Health, Dr. David Butler-Jones (PHAC) and the Federal Minister of Health Leona Aglukkaq, only appeared in 2% of clips to explain the vaccine shortage and the initial characterization of H1N1 as a Phase 6 pandemic. Members of the public received 10.1% of audio airtime in interviews and 11.2% of visual airtime, while newscasters received the vast majority of both audio (69.4%) and visual (29.7%) airtime.

Federal and provincial public health experts were portrayed wearing suits and ties in 92% of clips; none were portrayed as practicing health professionals in white coats or scrubs. Interviews were conducted in press conferences and government buildings rather than hospitals or vaccination clinics.

### Information Screens and Images

Static information screens (n = 31) displayed information in conjunction with audio content, predominantly for the results of surveys, mortality and hospitalization statistics, as well as flu symptoms. The screens were dark blue with yellow accents and used medical symbols, including: the caduceus; electrocardiogram readouts; a petri dish streaked with red cultures; microscope images of bacteria; and a bottle with a syringe. An association was made between the petri dish, bacterial and cell cultures and the H1N1 pandemic in 62% of screens.

### Interviews with Health Reporters

The media experts we interviewed in this study identified logistical challenges that limited the footage available to editors for broadcast. Stock footage was reused in part because of access restrictions for filming in hospitals and clinics. With rapidly developing stories, it was difficult for reporters to access interviewees and locations in time to meet deadlines. The re-use of lead footage and visuals was a concession to practicality as editors attempted to meet the demands of a 24-hour news cycle. The information provided by the interviewees confirmed that, in their opinion, our overall findings with respect to CTV were generalizable to television news coverage more broadly.

## Discussion

A report by the Health Quality Council of Alberta found that “dealing with the media became a significant issue and challenge” [8] for the public health workers and identified the negative responses of the media to government decisions communicated by public health agencies as a significant concern for public health workers [8]. A review of the response of Alberta Health and Wellness to the H1N1 pandemic found that there was confusion about which agencies should speak to the media and what information should be released to the public [23]. Our results suggest that visual media posed additional challenges addressed by neither report.

CTV coverage focused on failures in the vaccination campaign, evidenced by long line-ups and nearly daily early closures at the clinics, followed by the cancellation of all clinics October 31, 2009 because of an unanticipated shortage of the vaccine. Canadian health news often focuses on such conflicts in health care delivery and tends to hold the government responsible for public health troubles, rather than individuals [24]. The use of this conflict frame is problematic as it increases public cynicism [25]. Our study confirmed the use of the conflict frame as television visuals emphasized the disorganized and confused official response to the H1N1 pandemic. This ran counter to the principle that demonstrating the convenience of getting vaccinated encourages vaccination behaviour [26]. Indeed, the negative portrayal of the organization and rollout of the vaccination campaign was a form of anti-vaccination message. Even infrequent anti-vaccination stances in the media are related with non-compliance [7]. At the end of the vaccination campaign, only 36.6% of Albertans had been vaccinated, less than the national vaccination rate of 41.3% [10].

### Confusion Over the Vaccination Rollout

Media reflect the confusion of public health agencies, augmenting public confusion [27]. Our results show several aspects of local news reporting that may have contributed to public confusion. Initially, Alberta’s public health agencies tried to convince people to get vaccinated. Then, on October 30, 2009, CTV interviewed Minister Liepert, who said, “I don’t have answers for [Albertans] … We will be out of vaccine by early next week.” This about-face from initial assurances of sufficient vaccine caused a rush to the vaccination clinics, which resulted in long line-ups and frustrated citizens.

Countering the message that all Albertans should be vaccinated, CTV showed results of polls suggesting about half of Canadians were not planning to get the vaccine, echoing poll results from the United States [28]. In addition, public health agencies identified priority groups, which is a common tool in the arsenal of public health agencies for interventions like vaccination during a pandemic. Other studies have found that advertising priority groups reinforces behaviour change and health behaviours including vaccination [29] among priority group members. However, it also decreases the risk perception of individuals not identified as priority groups [29].

Our results show that communications targeted towards identifying priority groups to enhance the efficiency of the vaccination rollout were equally confusing. Efforts to encourage vaccination behaviour by showing healthy individuals getting vaccinated directly contradicted audio content about priority groups. Research shows that television viewers pay more attention to the news than other programming [30], [31], but does not show whether busy viewers pay more attention to the audio content or the visual content. If viewers privileged one modality over the other, Albertans were likely left with two very different impressions of who should get vaccinated, when.

Alberta’s public health agencies emphasized the importance of vaccinating certain groups, but themes of potential interest to priority groups, such as pregnant women (e.g. comparisons of adjuvanted and non-adjuvanted vaccine), were uncommon on television news, dominated instead by the long line-ups at vaccination clinics. Nevertheless, no matter what public health officials sought to communicate about best practices for the vaccination rollout, many of the decisions, which resulted in visual-audio discrepancies in news broadcasts as well as discrepancies between content and public health agency messages, were taken by the media, namely, news producers and journalists. This emphasizes the need for public health agencies to better understand the needs and operational constraints of their communications partners, in this context, the television news.

Studies from other jurisdictions, namely the United Kingdom and Australia, show that media may be effective partners in communicating information about mass vaccination campaigns [32], [33]. In these countries, there was surprisingly little hype about the pandemic, at least in the print media, a finding mirrored by one study in Canada [7]. Nevertheless, while abundantly studied, print media are not synonymous with online and television sources, and conclusions drawn from print media do not diminish the urgent need for public health agencies to develop partnership strategies with multiple news formats, reflecting the respective needs of different media.

### Trust in Public Health Officials

Confidence in public health authorities is necessary to motivate compliance with controversial recommendations [34], such as H1N1 vaccination. The public health officials who appeared on television were shown as government officials wearing suits and ties, not health professionals. They were also most frequently shown in formal press conference environments or with government buildings in the background, thus reinforcing a visual identity as ‘politicians’ or ‘bureaucrats’ instead of public health experts or professionals. This is significant because more Canadians trust doctors (78%) than trust politicians and bureaucrats [35], an international trend [36]–[38].

The public has long been known to attribute low credibility to government spokespersons based on many factors, including insensitivity to the information needs and concerns of the public [39]. A deliberative forum conducted in Australia to elicit community perspectives on communication during a pandemic influenza outbreak identified a public desire for trusted media spokespeople who are ‘experts’ rather than ‘politicians’ on the basis that “communication should not be used for political point scoring” [40]. In Canada, focus group participants agreed that they want traditional media pandemic information to come from senior health officials and scientists demonstrably involved in managing the crisis because they feel these people can provide the most accurate and complete information [41]. Participants specifically indicated that they do not want to receive information ‘second-hand’ from politicians or agency spokespeople [41]. One media interviewee in this study noted that during the SARS outbreak in Toronto, Dr. Sheela Basrur (then Toronto’s Medical Officer of Health) increased her credibility by appearing as a physician in a clinical setting. This “white coat effect” makes doctors appear more authoritative in communicating public health messages [42].

Failure to recognize the importance of appropriate spokespeople in crisis risk events has been shown to result in confusing messages and a loss of credibility [43]. Trust is engendered when people believe that decision-makers share their sense of values and have confidence in their past performance. The public is reassured by this sense of proximity to their decision-makers [44]. The bureaucratic setting of press conferences sharply demarcated Albertan officials from the public. The defensiveness of officials on previous decisions and statements, evidenced through the dominant theme of “official response” to prior criticism may have undermined Albertans’ trust even further. Interviewed media experts in this study suggested that press conferences were “out of step” with the requirements of the modern news media. To increase public confidence, public health agencies should use spokespeople who are clearly identified as local and relevant health professionals and as competent decision-makers.

### Information Screens: Colour and Symbolism

Information screens made up a part of the news visuals. Scientific images have two purposes: to visualize an unfamiliar topic and to encourage recall about the information [45]. Footage was often re-used due to production constraints, so medical imagery such as the caduceus and injection paraphernalia became visual shorthand to identify health-related news. Red was used in the petri dish image to convey alarm and danger [46]. Juxtaposition of human elements, like cells with dark, mechanical or alien elements, can contribute to negative valence toward the technical elements [45]. Public health agencies need to be more aware of the emotive effects of colour and symbolism in visual media to encourage positive health behaviours and discourage panic.

### Limitations and Other Considerations

The main limitation of this study is that it analysed television coverage on one high profile television station in in a single Canadian province, Alberta. Nevertheless, CTV is a national network and attracts a large viewership for its local evening news programs, which has a consistent format across the country, making these results transferable to other areas in Canada. While Canadians may also be exposed to news on multiple cable and satellite channels, these are international and do not focus on Canadian or local news. In addition, our analyses and conclusions were supported by the media experts we interviewed, who discussed these issues as not being unique to CTV news.

We also acknowledge that television news viewing may sometimes be incidental or accidental, while at other times it may be purposeful and information-seeking. We did not examine how Albertans understood or used the television news about H1N1 and the vaccination campaign in their decision-making processes related to vaccination. However, a number of studies found that people who were dependent on television for H1N1 and vaccine information were less likely to be vaccinated [47]–[49]. In our study this effect may have been because television included more statements reflecting public distrust than print media [50] and because television made greater use of exemplars from the public (e.g., through interviews and visuals of members of the public in our study) [51]. We found that such interviewees predominantly expressed frustration with the vaccination rollout and long line-ups.

Finally, our study did not examine all the factors that contributed to low vaccination rates in Alberta. Other studies have shown a multiplicity of reasons for not being vaccinated, predominantly (1) not believing H1N1 presented a significant risk [52]–[54] and (2) not feeling the vaccination was safe [52]–[54]. Even individuals who believed that H1N1 was a significant health risk avoided vaccination over safety concerns [55]. For these individuals, information sources were unable to mitigate their concerns about vaccine safety [55]. Finally, vaccine safety concerns impacted the vaccination choices of parents, many of whom were more willing to accept the risk of H1N1 to their children than an adverse response to the vaccine [56].

While there was certainly concern in Alberta, as in other jurisdictions, over the safety of the vaccine, the initial rush to the vaccination clinics was indicative of high levels of intention to be vaccinated [8]. The chaotic rollout turned many away from vaccination at the clinics [8], and this message of chaos and frustration with public health officials was reflected in the news media [8]. Indeed the 2010 report from the Health Quality Council of Alberta concluded that fewer people than expected were vaccinated in Alberta during the pandemic, leaving 1,017,926 unused doses at the end of the vaccination campaign. This was due to two factors: (1) Alberta Health and Wellness and Alberta Health Services initially chose not to vaccinate high-risk groups first; and (2) the agencies were not flexible enough to deal with higher-than-anticipated initial demand or problems like vaccine shortages because the choice of venues and staffing decisions could not accommodate the crowds that showed up in the first week [8].

### Conclusion

While this study analysed television coverage within a single province, the communication challenges faced by Alberta’s public health agencies were not isolated and extended across other provinces and to the Public Health Agency of Canada. Our analysis of CTV news coverage of the 2009 H1N1 pandemic highlights some key lessons for all public health agencies, including the need to partner effectively with local media outlets, such as television stations, to develop clear, consistent and credible communications. Most importantly, public health agency communications strategies must account for the dual format of television news and provide ready-to-use visual content to complement key messages. In 2009, the news footage did not match the main public health messages delivered from sterile and staged environments so that the messages about risk levels and at-risk groups were unclear and inconsistent. Public health agencies clearly lost control of their message about preventive measures for the H1N1, most importantly, vaccination. Instead, the media focussed on the disorganized vaccine rollout, shortages and long line-ups. Public health spokespeople need to be well-trained, confident and accessible to the media. In the future, local credibility may be enhanced with spokespeople emphasizing their role as public health professionals and appearing in clinical settings or interacting directly with the public. Public health agencies need to learn from the H1N1 pandemic so that television communications do not add to public confusion [57], demonstrate bureaucratic ineffectiveness and possibly contribute to low vaccination rates.

## Supporting Information

Table S1 Publically available sources for CTV news clips*.
(DOCX)

Table S2 Codebook for audio content with codes and definitions.
(DOCX)

Table S3 Codebook for visual content with codes and descriptions.
(DOCX)

Table S4 Codebook for content and graphics of static information screens with codes and descriptions.
(DOCX)
