# Supplementary material for: When Pictures Waste a Thousand Words: Analysis of the 2009 H1N1 Pandemic on Television News
Source: PLoS One. 2013 May 17;8(5):e64070. doi: 10.1371/journal.pone.0064070 (PMC3656930; doi:10.1371/journal.pone.0064070)
Supplement: Table S1 — Publically available sources for CTV news clips*. (DOCX) [file pone.0064070.s001.docx]

Table S1. Publically available sources for CTV news clips*.

| **URL** |
| --- |
| http://www.ctvnews.ca/video?clipId=232055 |
| http://www.ctvnews.ca/video?clipId=231843 |
| http://www.ctvnews.ca/video?clipId=231040 |
| http://www.ctvnews.ca/video?clipId=231039 |
| http://www.ctvnews.ca/video?clipId=231035 |
| http://www.ctvnews.ca/video?clipId=230040 |
| http://www.ctvnews.ca/video?clipId=230032 |
| http://www.ctvnews.ca/video?clipId=229270 |
| http://www.ctvnews.ca/video?clipId=229022 |
| http://www.ctvnews.ca/video?clipId=229017 |
| http://www.ctvnews.ca/video?clipId=228776 |
| http://www.ctvnews.ca/video?clipId=228557 |
| http://www.ctvnews.ca/video?clipId=228547 |
| http://www.ctvnews.ca/video?clipId=228306 |
| http://www.ctvnews.ca/video?clipId=182244 |
| http://www.ctvnews.ca/rush-for-vaccine-puts-high-risk-groups-at-risk-experts-1.448144 |
| http://www.ctv.ca/CTVNews/World/20091105/vaccine_sideeffects_091105/ |
| http://www.ctv.ca/CTVNews/TopStories/20091109/H1N1_peak_091109/ |
| http://www.ctv.ca/CTVNews/TopStories/20091105/spread_091103/ |
| http://www.ctv.ca/CTVNews/Politics/20091029/vaccine_debate_091029/ |
| http://www.ctv.ca/CTVNews/Politics/20090510/EDM_pigs_090510/ |
| http://www.ctv.ca/CTVNews/MSNHome/20090430/alta_flu_090430/ |
| http://www.ctv.ca/CTVNews/Health/20091026/flu_ads_091026/ |
| http://www.ctv.ca/CTVNews/EdmontonHome/20091030/vaccine_flow_091030/ |
| http://www.ctv.ca/CTVNews/CanadaAM/20091101/h1n1_confusion_091101/ |
| http://www.ctv.ca/CTVNews/CanadaAM/20091026/swine_rollout_091027/ |

*Some links are to pages with several videos embedded in a java player. All links were active on March 3, 2013.
