# Supplementary material for: When Pictures Waste a Thousand Words: Analysis of the 2009 H1N1 Pandemic on Television News
Source: PLoS One. 2013 May 17;8(5):e64070. doi: 10.1371/journal.pone.0064070 (PMC3656930; doi:10.1371/journal.pone.0064070)
Supplement: Table S2 — Codebook for audio content with codes and definitions. (DOCX) [file pone.0064070.s002.docx]

| Table S2. Codebook for audio content with codes and definitions. | |
| --- | --- |
| **Audio Content Codes** | **Description** |
| Adjuvant or Non-adjuvant | Discussion of the H1N1 vaccine as adjuvant or non-adjuvant and reference to adjuvant in vaccine safety for pregnant women |
| Clinics shut (early) | The closure of clinics earlier in the day than planned |
| Clinics shut (temporary) | The closure of vaccination clinics in Alberta between October 31 and November 5, 2009 |
| Criticism of government | Implicit and explicit criticism of the pandemic response, the organization of the vaccination clinics, the vaccination process, etc. |
| Doctors and pharmacists | The role of doctors and pharmacists in administering the H1N1vaccine |
| Flu assessment clinic | The flu assessment clinics, which opened October 29, 2009 to relieve burden on emergency rooms |
| Flu impacts on businesses | The flu or the flu clinics impacting business operations |
| Flu information | Symptoms, transmission, self-care |
| Frustration | Expressions of frustration (e.g., “I’m frustrated with this line-up”) or frustrated tone identified during speech |
| Group-specific interests | Identification of groups that were not priority groups but felt that they should have early access to vaccination |
| Group-specific interests : Calgary Flames | Coverage of the Calgary Flames (ice hockey team) as a specific group who got access to the vaccine outside of the public vaccination clinics |
| Queue jumpers | Preferential treatment, jumping the queue or avoiding waiting in line-ups |
| Line-ups | Line-ups or queues, waiting for the vaccine and similar. |
| Name change | Swine flu to H1N1 |
| New cases | Identification of novel cases of H1N1 |
| Pandemic | Characterization H1N1 as a widespread, global or national issue. Identifying when H1N1 is treated as greater-than-local phenomenon. |
| Priority groups | AHW*-defined priority groups** and groups identified in the media as priority groups even though they were not according to AHW |
| Priority groups/Chronically ill | All mentions of chronically ill |
| Priority groups/Chronically ill/Children under 10 with chronic health conditions | AHW priority group of children under 10 with chronic health conditions |
| Priority groups/Chronically ill/Seniors under 65 with chronic health conditions | AHW priority group of seniors under 65 with chronic health conditions |
| Priority groups/Chronically ill/unspecified | Mentions of chronically ill outside of above-given age ranges |
| Priority groups/Front line health workers | AHW priority group of front line health care workers |
| Priority groups/Infants | Equivalent to AHW priority group 6 months to 5 years. |
| Priority Groups/Parents of babies under 6 months | As caretakers of dependents who were priority group members |
| Priority Groups/People in remote communities | AHW priority group of people living in remote communities |
| Priority Groups/People who care for high-risk individuals | AHW priority group of caretakers of high-risk individuals |
| Priority Groups/Pregnant women | Included references to pregnant women and foetus |
| Priority Groups/Seniors | Age ranges not given in media. Not a high AHW priority group unless chronically ill |
| Priority Groups/Young children | Age ranges not given in media. Not a high AHW priority group unless chronically ill |
| Reopening of clinics | References to the reopening of clinics to priority group November 5, 2009 |
| Response from officials | Responses by health or government officials to criticism or events previously identified in the newscast |
| Schools | Schools being closed, schools as a place of high vaccination, school children or policy related to schools |
| To get the vaccine or not | Vaccine safety, pros and cons of vaccination versus danger posed by H1N1 |
| Vaccination | Vaccine, vaccination, vaccine clinic |
| Vaccine Shipments | Future shipments of vaccine to Alberta, delays to shipments, expected dates of shipments of vaccine |
| Vaccine Shortage | Shipments that are lower than expected, and shortages that led to vaccine clinic closures in Alberta |

*Alberta Health and Wellness.

** Alberta Health and Wellness [11].
