# Supplementary material for: When Pictures Waste a Thousand Words: Analysis of the 2009 H1N1 Pandemic on Television News
Source: PLoS One. 2013 May 17;8(5):e64070. doi: 10.1371/journal.pone.0064070 (PMC3656930; doi:10.1371/journal.pone.0064070)
Supplement: Table S3 — Codebook for visual content with codes and descriptions. (DOCX) [file pone.0064070.s003.docx]

| Table S3. Codebook for visual content with codes and descriptions. | |
| --- | --- |
| **Visual content code** | **Description** |
| Depictions of medical authority | People who are associated with AHW*, AHS**, PHAC*** and Health Canada who were providing information to the public or who appear to be associated with the pandemic response plan |
| **Depictions of medical authority/Appearance** | **Visual characteristics of the interviewee** |
| Depictions of medical authority/Appearance/doctor | Male or female wearing scrubs, white doctor’s jacket, etc. |
| Depictions of medical authority/appearance/female, casual clothing | Female, wearing casual clothing (less formal than a suit) |
| Depictions of medical authority/appearance/female, suit | Female, wearing a suit or other professional attire equivalent to a suit |
| Depictions of medical authority/appearance/male suits, no tie | Male, wearing a suit without a tie. Top button may be done up or left undone |
| Depictions of medical authority/appearance/male, no suit, tie | Male, without suit jacket, wearing buttoned shirt and tie |
| Depictions of medical authority/appearance/male, shirt, no tie | Male, without suit jacket, buttoned shirt, no tie |
| Depictions of medical authority/appearance/male, suit + tie | Male, wearing full suit and tie |
| Depictions of medical authority/appearance/poppy | Appearance of a poppy (red flower with black or green centre) in lapel, commonly worn around November 11 to commemorate Remembrance Day |
| Depictions of medical authority/Important people | Key people in the communication strategy – top health officials |
| Depictions of medical authority/important people/Dr. Andre Corriveau | Alberta’s Chief Medical Officer of Health (AHS) |
| Depictions of medical authority/important people/Dr. Butler-Jones | Chief Public Health Officer of Canada (head of PHAC) |
| Depictions of medical authority/important people/Dr. Gerry Predy | Senior Medical Officer of Health (AHS) |

| Depictions of medical authority/important people/Dr. Judy Macdonald | Calgary Zone Medical Officer of Health (AHS) |
| --- | --- |
| Depictions of medical authority/important people/Dr. Marcia Johnson | Edmonton Zone Medical Officer of Health (AHS) |
| Depictions of medical authority/important people/Leona Aglukkaq | The Honourable Minister of Health (Health Canada) |
| Depictions of medical authority/important people/Ron Liepert | Alberta Minister of Health (head of AHW) |
| Depictions of medical authority/important people/Steven Duckett | President and CEO of AHS |
| **Depictions of medical authority/Places** | **The physical location of the interviews with people in positions of medical authority** |
| Depictions of medical authority/places/at the clinics | Person is at a vaccination clinic |
| Depictions of medical authority/places/doctor office | Person is in a doctor’s office or examining room |
| Depictions of medical authority/places/generic | Interviews that occur in places without discernible distinguishing features |
| Depictions of medical authority/places/government buildings | Person is in legislative chamber, or other building/room/hallway with crest of government visible |
| Depictions of medical authority/places/office | Person is in an office. Distinguished from a government space if lacks distinguishing logos, crests or other visual cues associated with government |
| Depictions of medical authority/places/outside | Person is outside, not in a building or other structure |
| Depictions of medical authority/places/portrait | Footage of person is not used. A static picture is shown |
| Depictions of medical authority/places/press conference | Person behind a table, with a microphone sitting in a room facing members of the media. Possibly agency or government name/logo on backdrop behind speaker |
| Depictions of medical authority/places/voice over | Absence of a person of medical authority in footage while their voice is present in audio |
| **Interviewed persons** | **People interviewed by the media** |
| Interviewed persons/characteristics | Characteristics of interviewed people that were used to identify them as priority group members |
| Interviewed persons/characteristics/bad experience | People having a negative experience with H1N1 or the vaccination clinics identified by facial expression and body language |
| Interviewed persons/characteristics/business impacted by flu or vaccine | People whose businesses have been impacted by H1N1, the vaccination clinics or other public health responses to H1N1 |
| Interviewed persons/characteristics/children | People who appear to be younger than 18 |
| Interviewed persons/characteristics/health care worker | People who appear to be health care providers through cues like scrubs, EMS uniforms, white doctor’s coat, other health paraphernalia |
| Interviewed persons/characteristics/native | People who appear to be of aboriginal descent |
| Interviewed persons/characteristics/not in a priority group | People who do not appear to fit into any of the groups defined in other categories |
| Interviewed persons/characteristics/parent | Person in the vicinity of a child during the interview or who were identifiable as parents by vicinity to child-related items (strollers, backpacks, toys, etc.) |
| Interviewed persons/characteristics/possible government official | People who appear to be officials, but are not visually identified with government, AHW or another involved agency |
| Interviewed persons/characteristics/senior | Persons appearing older than 50. Subjective, based on hair/facial features/etc. |
| Interviewed persons/female interviewee | Women/girls interviewed by the media |
| Interviewed persons/male interviewee | Men/boys interviewed by the media |
| **Other authority** | **Other people who do not appear to be a part of the general public in their appearance, setting or in the content of their speech. May include government officials, subject experts like professors, analysts, etc.** |
| Other authority/at clinics | Person is at a vaccination clinic or in a line associated with a vaccination clinic |
| Other authority/casual | Person is wearing casual attire |
| Other authority/government | Person who appeared to be in government because of title, setting or bearing |
| Other authority/government/ Brian Mason | Then- leader of the New Democratic (NDP) Party |
| Other authority/government/ David Swann | Then- leader of the official opposition in Alberta (Alberta Liberal Party) |
| Other authority/government/ Ed Stelmach | Then-Premier of Alberta (Alberta Progressive Conservative Party leader) |
| Other authority/government spaces | Person is in legislative chamber, or other building/room/hallway with crest of government visible |
| Other authority/home | Person is in a homey setting, suggested by personal belongings, furniture style |
| Other authority/non-government authority | People who appear to have some authority, but are not members of municipal, Albertan or Canadian government |
| Other authority/poppy | Appearance of a poppy (red flower with black or green center) in lapel, commonly worn around November 11 to commemorate remembrance day |
| Other authority/suit and tie | People wearing formal attire, including suit and tie or gender/appropriate equivalent |
| **Visual Codes - Other** |  |
| Emergency Medical Services | Depictions or interviews of people in Emergency Medical Services uniforms |
| Getting the shot | Depictions of individuals getting a needle or sitting in a chair being prepped for a needle |
| Other than vaccine preventative measures | Visuals of other recommended behaviours like sneezing into one’s sleeve |
| Pregnant women | Visuals of pregnant women |
| Line-up visuals | Footage with people in queues/line-ups |
| No line-ups | Visuals of a lack of line-ups/queues in places where occurred in prior video clips |
| Information screen (note that Information screens were coded separately for symbols and content) | Screen featuring text with information on H1N1, the vaccine, priority groups, etc. |

*Alberta Health and Wellness

**Alberta Health Services

***Public Health Agency of Canada
