# Supplementary material for: When Pictures Waste a Thousand Words: Analysis of the 2009 H1N1 Pandemic on Television News
Source: PLoS One. 2013 May 17;8(5):e64070. doi: 10.1371/journal.pone.0064070 (PMC3656930; doi:10.1371/journal.pone.0064070)
Supplement: Table S4 — Codebook for content and graphics of static information screens with codes and descriptions. (DOCX) [file pone.0064070.s004.docx]

Table S4. Codebook for content and graphics of static information screens with codes and descriptions.

| **Code** | **Description** |
| --- | --- |
| Information screen | Screen featuring text with information on H1N1, the vaccine, priority groups, etc. |
| Information screen/content/fatalities | Information screens with the number of people whose death was attributed to H1N1 |
| Information screen/content/cases | Information screens with the number of new, current or total cases of H1N1 |
| Information screen/content/symptoms | Information screen with H1N1 symptoms |
| Information screen/content/vaccine information | Information screen with vaccine ingredients, safety information, etc. |
| Information screen/content/priority groups | Information screen identifying priority groups for vaccination |
| Information screen/content/clinic information | Information screens with information on the hours and/or locations of H1N1 vaccination clinics |
| Information screen/content/poll results | Information screens showing the results of polls about H1N1 |
| Information screen/images/petri dish with bacteria culture | A magenta-coloured petri dish with horizontal and vertical bacteria streaks in sickly yellow |
| Information screen/images/bacteria picture | Microscope image of a group of pale blue-green single cells, irregularly shaped. |
| Information screen/images/needle | Images of a hypodermic needle |
| Information screen/images/surgical glove | Images of a hand in a green surgical glove usually holding a hypodermic needle |
| Information screen/images/Alberta | Image with an outline of Alberta |
| Information screen/images/caduceus | Image of a caduceus (staff with wings entwined by two serpents) |
| Information screen/images/Vitruvian man | Image of the Vitruvian Man (drawing by Leonardo da Vinci with two superimposed drawings of a man with his arms and legs outstretched inscribed inside square and a circle) |
| Information screen/images/EKG output | Electrocardiography (ECG or EKG) output with regular peaks and valleys |
| Information screen/images/Map of an H1N1 event | Map of Alberta showing where an event occurred in relation to nearby cities and towns |
| Information screen/blue background | Information screen has a blue background |
| Information screen/blue-yellow background | Information screen has a blue and yellow background |
